# Data related to measures of physiological arousal during everyday life experiences and their relation to self-reports of subjective experience of both the event and its memory

**DOI:** 10.1016/j.dib.2019.104823

**Published:** 2019-11-16

**Authors:** Sinué Salgado, Osman Skjold Kingo

**Affiliations:** Aarhus University, Department of Psychology, Center on Autobiographical Memory Research, Denmark

**Keywords:** Emotion, Physiology, Mindfulness, Autobiographical memory, Self-reports

## Abstract

Emotionally intense experiences lead to particularly durable and detailed autobiographical memories (AM) [1,2]. However, the influence of arousal on self-reports of the phenomenological characteristics of events and AMs is not direct, but moderated at the cognitive level [3,4]. To address how individual differences in emotional awareness moderate the physiology-subjective experience link, we collected data using a questionnaire from the mindfulness literature, the Five Facet Mindfulness Questionnaire (FFMQ [5]). In addition, objective measures of physiological arousal while events naturally unfolded in everyday life contexts—outside the lab—were collected to map them onto self-reports of their phenomenological characteristics and those of their memories. In this article, we provide the full data for the FFMQ from a sample of 60 undergraduate students. We also display analyzed data of how markers of physiological arousal (i.e., electro-dermal activity, heart rate, and temperature) related to self-reports at two time-points of interest. First, we related these measures to same day self-reports of the characteristics of the experience. Then, we related these measures to self-reports and to arousal of their memories one week later, and in the lab. Detailed interpretation of this data, as well as in depth theoretical background is presented in “How is physiological arousal related to self-reported measures of emotional intensity and valence of events and their autobiographical memories?” [6].

**Table:** Specifications Table

Subject area	*Psychology*
More specific subject area	*Autobiographical Memory*
Type of data	*Tables*
How data was acquired	*Biomarkers of arousal were collected using a wristband (Empatica E4). Self-reports were collected through questionnaires.*
Data format	*Raw, Edited & Analyzed data*
Experimental factors	*It was a within-subjects factorial design. Participants experienced and reported events in their normal everyday life while markers of physiological arousal where sampled. One week later, participants were cued for the events reported while also sampling their physiological arousal.*
Experimental features	*Outcomes measures were subjective self-reports of the characteristics of the experience as well as both arousal and self-reports for memories. We also measured individual differences such as emotional awareness.*
Data source location	*Aarhus, Denmark (Aarhus University)*
Data accessibility	*Full data for the FFMQ is with this article. Analyzed physiological data is presented in the body of the article. The raw physiological data will be shared by means of individual contracts of confidentiality by contacting the first author.*
Related research article	*Salgado, S. & Kingo, O. S. How is physiological arousal related to self-reported measures of emotional intensity and valence of events and their autobiographical memories? Consciousness and Cognition, 75, 2019, 102811* [6]

**Table:** 

**Value of the Data** • The variety of measures taken can make this data set a point of reference to further improve designs looking at Autobiographical Memory in light of individual differences. • Values for the degree of association between arousal taken during real life experience along their self-reports can be compared to similar designs in the lab associating arousal with subjective experience of emotion. • The associations presented here between objective markers of arousal and self-reports can be used as a reference for studies in the Autobiographical Memory field, which has largely relied on subjective evaluations.

## 1 Data

**Fig. 1:**
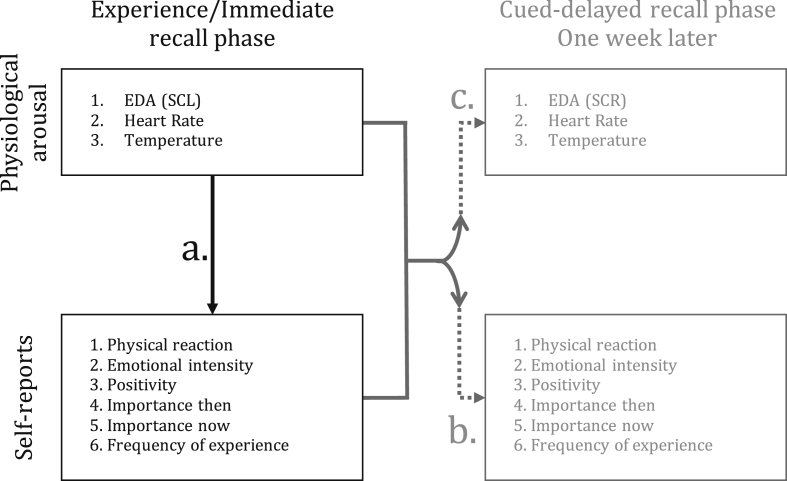
Schematic representation of the planned analyses for the data collected. The association illustrated by a. Represents the relation between physiological arousal as the events unfold with their self-reports in the diary. The association in b. Illustrates the relation between both physiological arousal while the event unfolded and its self-reports—together— with the characteristics of the memory in the lab. Similarly, c. Illustrates the relation between both events' physiology and self-reports—together—with the memories' physiological measures of arousal. For the experience/immediate recall phase the skin conductance level (SCL) was preferred as the measure for EDA. As for the cued-delayed recall phase, skin conductance reaction (SCR) was the selected measure for EDA.

This article shows the full raw data for the five factors of the FFMQ assessing individual differences in mindfulness components skills (Table 1). In addition, Fig. 1 illustrates the planned analyses for the collected data pertaining to both physiological data and data of self-reports. We present correlations between objective measures of arousal during everyday life experiences and the subjective evaluations of their characteristics (Table 2). Also, the correlations of physiological arousal during the events with both self-reported characteristics of their memories (Table 3) and measures of arousal while recalling the events one week later are displayed (Table 4). We do so by presenting the values for each step of three hierarchical multiple regression analyses. These analyses were carried on as to predict self-reports of the experience, self-reports of the memory, and the arousal during memory recall from the measures of arousal as events unfolded. As biological markers of physiological arousal fall under the category of personal sensitive information under the European Union General Data Protection Regulation (GDPR), we cannot publicly display—either here or in any other public repository—the raw data of our measures of arousal. However, it is possible to share the raw physiological data by means of individual contracts of confidentiality by contacting the first author.

**Table 1:** Added values/scores for each of the five factors in the Five Facet Mindfulness Questionnaire as a function participant.

	Five Factors				
	Observe	Describe	Act with awareness	Nonjudge	NonReact
1	29	25	27	27	13
2	30	24	22	19	22
3	27	26	30	27	30
4	21	33	25	34	25
5	30	25	28	24	15
6	28	29	28	34	23
7	26	32	16	29	26
8	28	32	24	24	12
9	30	30	29	21	27
10	26	26	22	25	20
11	30	37	24	25	17
12	26	35	34	39	19
13	24	20	24	29	17
14	29	38	29	40	25
15	22	37	20	23	23
16	25	30	19	26	18
17	30	34	24	36	15
18	21	34	32	32	24
19	24	32	16	32	18
20	34	21	15	20	20
21	30	24	19	18	19
22	40	36	38	37	21
23	35	37	22	34	27
24	33	33	23	26	19
25	31	39	34	37	30
26	33	25	28	34	26
27	30	35	32	34	27
28	24	38	33	23	25
29	25	37	29	34	25
30	29	29	21	27	16
31	28	29	26	38	24
32	37	32	22	33	28
33	37	28	27	18	24
34	26	25	35	29	21
35	28	26	32	33	26
36	18	36	31	39	32
37	24	30	20	23	14
38	28	32	30	37	21
39	34	27	22	33	22
40	35	27	30	24	20
41	29	33	19	17	17
42	30	27	25	31	22
43	20	30	37	30	22
44	23	32	31	35	19
45	30	25	28	26	21
46	25	27	30	30	18
47	21	34	28	36	16
48	29	30	20	39	26
49	32	23	39	27	27
50	32	39	21	13	20
51	28	31	21	17	16
52	29	39	32	22	20
53	37	40	21	37	20
54	24	27	28	36	31
55	27	29	28	36	15
56	30	21	19	34	25
57	28	31	27	33	22
58	32	33	29	29	24
59	36	33	34	24	24
60	30	35	32	32	25

*Note.* Possible range of scores for each factor is 8–40 (8 items each). The exception is the NonReac factor for which the possible range of scores is 8–35 (7 items).

**Table 2:** Hierarchical multiple regression analyses predicting each of the phenomenological characteristics of events that participants reported in their diary from objective measures of physiological arousal.

Predictors	Physical reaction		Emotional intensity		Emotional valence		Importance at experiencing		Importance at reporting		Frequency of experience	
	Δ*R*^*2*^	β	Δ*R*^*2*^	β	Δ*R*^*2*^	β	Δ*R*^*2*^	β	Δ*R*^*2*^	β	Δ*R*^*2*^	β
Step 1	.22***		.21***		.15		.26***		.25***		.20***	
Control variables												
Step 2	.06***		.00		.01		.01		.01		.01	
EDA (SCL)		.08		-.03		.06		.02		-.01		-.06
Temp		.17		-.01		-.09		-.12		-.09		.10
Heart Rate		.27***		.04		.02		.08		.06		.03
Total *R*^2^	.29***		.22***		.16		.27***		.26***		.21***	
n	439		439		439		439		439		439	

*Note*. *N* = 439 for analyses on the experience/immediate recall phase, and *N* = 290 for analyses on the cued-delayed recall phase. The variation on sample sizes is due to some participants not having data on either their physiological measures for some events or enough pictures to generate cues in the cued-delayed recall phase.

*p < .01, **p < .004, ***p < .001.

**Table 3:** Hierarchical multiple regression analyses predicting each of the phenomenological characteristics of the memories cued in the cued-delayed recall phase from both objective measures of physiological arousal and from self-assessed characteristics of the events.

Predictors	Physical Reaction		Emotional Intensity		Emotional Valence		Reliving		Importance at retrieving	
	Δ*R*^*2*^	β	Δ*R*^*2*^	β	Δ*R*^*2*^	β	Δ*R*^*2*^	β	Δ*R*^*2*^	β
Step 1	.23**		.28***		.20		.43***		.37***	
Control variables										
Step 2	.10***		.02		.02		.01		.05***	
EDA (SCL)		.26***		.06		.00		.10		.11
Temp		-.10		-.24		-.14		-.14		-.33***
Heart Rate		.21***		.04		.14		.01		.05
Step 3	.11***		.19***		.28***		.09***		.18***	
EDA (SCL)		.23***		.08		-.03		.09		.10
Temp		-.11		-.12		-.01		-.06		-.23**
Heart Rate		.12		.07		.17**		.02		.04
Physical reaction		.35***		-.10		-.08		.01		.02
Emotional intensity		-.10		.17*		.18*		-.01		.05
Emotional valence		.08		.16*		.46***		.19**		.24***
Importance at experiencing		.18		.30***		-.05		.19		.11
Importance at reporting		-.06		-.03		.12		-.02		.21
Frequency of experience		-.11		-.19***		-.17**		-.17**		-.13*
Total *R*^2^	.45***		.49***		.50***		.53***		.60***	
n	290		290		290		290		290	

*Note*. *N* = 439 for analyses on the experience/immediate recall phase, and *N* = 290 for analyses on the cued-delayed recall phase. The variation on sample sizes is due to some participants not having data on either their physiological measures for some events or enough pictures to generate cues in the cued-delayed recall phase.

*p < .01, **p < .004, ***p < .001.

**Table 4:** Hierarchical multiple regression analyses predicting each of the objective measures of arousal elicited by memories in the cued-delayed recall phase from both objective measures of physiological arousal and from self-assessed characteristics of the events.

Predictors	EDA (SCR)		Temperature		HR	
	Δ*R*^*2*^	β	Δ*R*^*2*^	β	Δ*R*^*2*^	β
Step 1	.65***		.96***		.85***	
Control variables						
Step 2	.00		.00		.00	
EDA (SCL)		.05		-.03		.07
Temp		.07		.03		.02
Heart Rate		-.02		-.02		-.03
Step 3	.02		.00		.01	
EDA (SCL)		.07		-.03		.06
Temp		.08		.03		-.01
Heart Rate		.00		-.02		-.06
Physical reaction		-.04		-.01		.10**
Emotional intensity		.11		.02		.05
Emotional valence		.04		-.01		.00
Importance at experiencing		.19		-.02		-.02
Importance at reporting		-.25**		.01		-.02
Frequency of experience		-.01		.00		.01
Total *R*^2^	.68***		.97***		.86***	
n	290		290		290	

*Note*. *N* = 439 for analyses on the experience/immediate recall phase, and *N* = 290 for analyses on the cued-delayed recall phase. The variation on sample sizes is due to some participants not having data on either their physiological measures for some events or enough pictures to generate cues in the cued-delayed recall phase.

*p < .01, **p < .004, ***p < .001.

## 2 Experimental design, materials and methods

Base on the premise that emotionally intense experiences lead to particularly durable and detailed autobiographical memories (AM) [1,2], data on everyday life experiences, their physiological measures of arousal, and self-reports was collected from 60 individuals across different study programs at the University of Aarhus, Denmark. The objective measures of physiological arousal were electro dermal activity (EDA), temperature, and heart rate (HR). They were collected using a wristband, the Empatica bracelet E4 [7]. The experimental design and methods are fully described in our article “How Is Physiological Arousal Related to Self-Reported Measures of Emotional Intensity and Valence of Events and Their Autobiographical Memories?” [6]. The latter also contains details about the selection of the FFMQ factor used as the measure of emotional awareness, in-depth description of the selection of events, and specifics of how the measures of physiological arousal were handled. Therefore, here we briefly explain only the essential procedure to understand the tables presented. Please see our paper for a full detail of the methods, discussion and interpretation of the results.

### 2.1 Design

The data collection was divided in two parts and over two weeks. Part One was called the “experiencing/same day recall phase”, and Part Two was referred as the “cued-delayed recall phase”. In the experiencing/same day recall phase, we were interested in examining how objective measures of physiological arousal while events unfolded in everyday life and ordinary contexts mapped onto self-reports of their phenomenological characteristics; (a) in Fig. 1 illustrates this relation. In the cued-delayed recall phase, we wanted to examine how both—and together—events’ physiological arousal and, as well, its self-evaluated phenomenological characteristics, influenced both the characteristics of the memory and its arousal while recalling it. These relations are correspondingly illustrated in (b) and (c) in Fig. 1.

### 2.2 Materials and methods

#### 2.2.1 Assessing individual differences

Knowing that the link between physiology and self-reports of subjective experience is moderated at the cognitive level [3,4]; we assessed individual differences in our sample during the first session with each participant and before starting data collection. We used a questionnaire from the mindfulness literature, the FFMQ [5], which measures the degree with which individuals are aware of their own thoughts and feelings as they unfolded. The FFMQ is a 39-item multifaceted scale covering five aspects of mindfulness. The five facets are: 1) observing, noticing, attending to sensations, perceptions, thoughts, and feelings (observe); 2) describing, labeling with words (describe); 3) acting with awareness, automatic pilot, concentration, non-distraction (actaware); 4) non-judging of experience (nonjudge), and; 5) non-reactivity to inner experience (nonreact). In a scale from 1 to 5 (1 never or very rarely true; 5 very often or always true), participants rated how true a series of statements describe their own experiences (e.g., When I'm walking, I deliberately notice the sensations of my body moving). The scores for each facet were calculated by summing up the response values of all items in each specific factor (reverse items were inverted prior to calculating scores). Higher scores on a facet reflects participants' higher agreement with the descriptions in the factor.

Cronbach's Alpha for the full 39 items of the FFMQ scale was 0.85. Overall, participants' scores on each of the five facets were higher than the median of possible range of scores. The possible range of scores were 8–40 for all the factors with the exception of the NonReact, which possible maximum score range up to 35. Participants scored on average 28.62 (SD = 4.61) for the observing facet; 30.73 (SD = 5.06) for describing; 26.52 (SD = 5.72) for acting with awareness; 29.35 (SD = 6.66) for non-judging of experience; and 21.77 (SD = 4.64) for non-reactivity to inner experience.

#### 2.2.2 Sampling of everyday life experiences, their physiological data, and self-reports

Participants came to our lab, filled out the FFMQ, and were instructed on the experience/same day report phase. They were instructed on how to use two devices. First, a small camera (the Narrative Clip) that automatically and silently triggered photographs from first-person perspective every 30 seconds. These photos were used in the second phase to cue the events the participants experienced during this first phase. The second device was the Empatica bracelet, designed to collect continuous and real-time physiological data in daily life. The Empatica wristband measured participants’ inter beat interval HR, EDA, and skin temperature. Participants were asked to wear these devices during the following three days over a weekend (Friday to Sunday). They also were instructed to keep a diary and to report the most emotionally intense experiences for each of the three days. Participants then reported and rated these events in a series of phenomenological characteristics at the end of each day.

To analyze how physiological markers of arousal—as each event unfolded—mapped onto the subjective evaluation of their characteristics, measures of EDA, HR and temperature were averaged for each event and threated as the independent variables in a series of analyses. Table 2 presents the steps in a series of hierarchical multiple regression analyses to predict each of the phenomenological characteristics of the events from their physiological markers. As the events were clustered around 60 participants, step 1 in the regression were sixty dummy variables to get rid of individual variance (see Ref. [8] for a similar procedure).

#### 2.2.3 Events’ physiology and self-reports and their relation to their memories

Participants returned to the laboratory 1 week later for *the cued-delayed recall phase*. In this phase, they were cued with the photos of the events they reported during *the experience/same day report phase*. Participants were tested individually, and before starting this session, they were asked to wear the Empatica wristband once more. Sitting in front of a computer's monitor, and using E-prime, each participant was presented with the photos of their events as to cue them to retrieve the memory of such experiences. After being cued, participants rated the phenomenological characteristics of each cued AM in the same scales with which they did one week before. All cues for the 3 days were randomized to avoid biases for more recent events.

The measures of physiological arousal as events unfolded and their self-reported characteristic in the *experience/same day report phase* were used together to predict the phenomenological characteristics of the cued memories in the lab. Table 3 presents the steps of a hierarchical multiple regression analysis to examine this. As before, step 1 in the regression accounted for individual variance.

We were also interested in examining how physiological arousal while the events unfolded together with their phenomenological characteristics during the *experience/same day report phase* related to the physiological arousal evoked when recalling them one week later. Similar to the previous analyses, we ran a series of hierarchical multiple regression analyses. We also controlled for individual differences in Step 1. The data related to each step of this analysis is presented in Table 4.

## Conflict of Interest

The authors declare that they have no known competing financial interests or personal relationships that could have appeared to influence the work reported in this paper.
